# Control of Virulence Gene Expression by the Master Regulator, CfaD, in the Prototypical Enterotoxigenic *Escherichia coli* Strain, H10407

**DOI:** 10.3389/fmicb.2017.01525

**Published:** 2017-08-11

**Authors:** Carla Hodson, Ji Yang, Dianna M. Hocking, Kristy Azzopardi, Qianyu Chen, Jessica K. Holien, Michael W. Parker, Marija Tauschek, Roy M. Robins-Browne

**Affiliations:** ^1^Department of Microbiology and Immunology, Peter Doherty Institute for Infection and Immunity, The University of Melbourne, Parkville VIC, Australia; ^2^Murdoch Childrens Research Institute, The Royal Children’s Hospital, Parkville VIC, Australia; ^3^Australian Cancer Research Foundation Rational Drug Discovery Centre, St. Vincent’s Institute of Medical Research, Fitzroy VIC, Australia; ^4^Department of Biochemistry and Molecular Biology, Bio21 Molecular Science and Biotechnology Institute, The University of Melbourne, Parkville VIC, Australia

**Keywords:** enterotoxigenic *E. coli*, CfaD regulon, virulence genes, transcriptional regulation, virulence inhibition

## Abstract

Enterotoxigenic *Escherichia coli* (ETEC) is the most common bacterial cause of diarrhea in children in developing countries, as well as in travelers to these countries. To cause disease, ETEC needs to produce a series of virulence proteins including enterotoxins, colonization factors and secretion pathways, which enable this pathogen to colonize the human small intestine and deliver enterotoxins to epithelial cells. Previously, a number of studies have demonstrated that CfaD, an AraC-like transcriptional regulator, plays a key role in virulence gene expression by ETEC. In this study, we carried out a transcriptomic analysis of ETEC strain, H10407, grown under different conditions, and determined the complete set of genes that are regulated by CfaD. In this way, we identified a number of new target genes, including *rnr-1*, *rnr-2*, *etpBAC*, *agn43*, *flu*, *traM* and ETEC_3214, whose expression is strongly activated by CfaD. Using promoter-*lacZ* reporters, primer extension and electrophoretic mobility shift assays, we characterized the CfaD-mediated activation of several selected target promoters. We also showed that the gut-associated environmental signal, sodium bicarbonate, stimulates CfaD-mediated upregulation of its virulence target operons. Finally, we screened a commercial small molecule library and identified a compound (CH-1) that specifically inhibited the regulatory function of CfaD, and by 2-D analoging, we identified a second inhibitor (CH-2) with greater potency.

## Introduction

Enterotoxigenic *Escherichia coli* (ETEC) is a leading cause of acute diarrhea in infants in developing countries and in travelers to these countries (Al-Abri et al., 2005; Paschke et al., 2011; Kotloff et al., 2013). The virulence hallmark of this pathogen is the ability to produce either one or both of two well-characterized enterotoxins: heat-labile (LT) and heat-stable (ST) enterotoxins (Croxen and Finlay, 2010). For successful infection, ETEC also requires the assistance of colonization factors, such as CFA/I, which allow the pathogen to adhere to the small intestinal epithelium (Gaastra and Svennerholm, 1996; Fleckenstein et al., 2010). The CFA/I fimbriae of the ETEC strain, H10407, are encoded by the *cfaABCE* operon, which is positively controlled by the transcriptional regulator, CfaD (Caron and Scott, 1990). In ETEC strains that produce CS1 or CS2 fimbriae (encoded by the *cooBACD* operon), the Rns protein (a CfaD homolog) activates their transcriptional expression (Caron et al., 1989). CfaD/Rns also activate the expression of CS4, CS14, CS17, and CS19 (Bodero and Munson, 2016). Collectively, ETEC strains bearing these fimbriae constitute approximately 80% of human isolates.

CfaD and Rns are members of the AraC family of transcriptional regulators and is closely related to other virulence regulators such as AggR from enteroaggregative *E. coli* (Nataro et al., 1994; Gallegos et al., 1997), ToxT from *Vibrio cholerae* (DiRita et al., 1991), RegA from *Citrobacter rodentium* (Yang et al., 2009) and VirF from *Shigella* species (Dorman, 1992). CfaD is a protein of 265 amino acids whose carboxy-terminal domain contains two helix-turn-helix (HTH) DNA-binding motifs. The amino-terminal domain of ToxT and RegA is implicated in dimerization and cofactor binding, but the function of the corresponding region of CfaD is unknown.

The promoter regions of the operons controlled by these regulatory proteins are generally AT-rich and exhibit a high degree of intrinsic DNA curvature (Dorman, 2007; Yang et al., 2011). The global regulator, H-NS, is able to bind to these sequences and silence their expression by blocking access of RNA polymerase to the promoters (Dorman, 2007; Yang et al., 2011). As with other AraC-like virulence regulators, CfaD activates transcription of its target operons by binding to asymmetrical, highly AT-rich sequences and displacing H-NS from the promoters (Gallegos et al., 1997). In addition to activating the *cfaABCE* operon, CfaD also activates the transcription of its own gene, and the *cexE* gene, which encodes a secreted protein homologous to the Aap dispersin of enteroaggregative *E. coli* (Pilonieta et al., 2007). Furthermore, CfaD also acts as a repressor of *nlp*, which encodes an inner membrane lipoprotein (Bodero et al., 2007). Although several studies have investigated CfaD-mediated regulation of specific target genes, no comprehensive characterization of the entire CfaD regulon has been carried out. In this study, we performed RNAseq transcriptomic analysis of the prototypical ETEC strain, H10407, and identified and characterized a number of previously unknown members of the CfaD regulon. The critical importance of CfaD in the control of ETEC virulence makes it a potential target for new types of drugs that could be used to prevent or treat ETEC infections. To test this possibility, we screened small molecule libraries for CfaD-specific inhibitors.

## Materials and Methods

### Bacterial Strains, Plasmids, Primers and Media

The bacterial strains and plasmids used in this study are listed in **Table 1**, and the primers are listed in **Table 2**. Bacteria were grown at 37°C in Luria-Bertani broth (LB) or on Luria-Bertani agar (LA) plates supplemented with antibiotics, when needed, at the following concentrations: ampicillin, 100 μg/ml; kanamycin, 50 μg/ml; trimethoprim, 40 μg/ml; chloramphenicol, 25 μg/ml.

**Table 1:** Strains and plasmids used in this study.

Strains/plasmids	Relevant characteristics	Source
***Escherichia coli* strains**		
H10407	Prototypical ETEC strain, O78:H11	Skerman et al., 1972
H10407Δ*cfaD*	Δ*cfaD*:: Kan^R^	This study
H10407Δ*cfaABCE*	Δ*cfaABCE*:: Kan^R^	This study
MC4100	F^-^*araD*139 (*argF-lac*) *lacU*169 *rpsL*150 *relA*1 *ΔbB*5301 *deoC*1 *ptsF*25 *rbsR thiA*	Casadaban, 1976
BL21(DE3)	F^-^ *ompT gal dcm lon hsdS_B_*(*r_B_*^-^*m_B_*^-^) λ(DE3 [*lacI lacUV5-T7p07 ind1 sam7 nin5*]) [*malB*^+^]_K-12_(λ^S^)	NEB
TOP10	F^-^ *mcrA* Δ(*mrr*^-^, *hsdRMS*^-^, *mcrBC*) ϕ80*lacZ* ΔM15 Δ*lacX*74 *nupG recA*1 *araD*139 Δ(*ara-leu*)7697 *galE*15 *galK*16 *rpsL*(Str^R^) *endA*1 λ^-^	Invitrogen
JP8042	Δ*lacU*169 *recA*56 *tyrR*366	Yang et al., 2013
**Plasmids**		
pGEM-T Easy	High-copy number vector, Ap^R^	Promega
pCR2.1-TOPO	High-copy number vector, Ap^R^, Kan^R^	Invitrogen
pACYC184	Medium-copy number vector, Cm^R^, Tc^R^	Chang and Cohen, 1978
pKD4	Vector containing Kan^R^ gene, Kan^R^, Ap^R^	Datsenko and Wanner, 2000
pKD46	Low-copy number vector, P_BAD_-λ Red, Ap^R^	Datsenko and Wanner, 2000
pMU2385	Single-copy number transcriptional fusion vector, Tp^R^	Yang et al., 2004
pMAL-c2x	Expression vector for N-terminal MBP-fusion protein, Ap^R^	NEB
pACYC184-CfaD	pACYC184 carrying the *cfaD* gene, Cm^R^	This study
pMAL-c2x-CfaD	pMAL-c2x carrying the *cfaD* coding region, Ap^R^	This study
*rnr-1-lacZ* (A)	*rnr-1* promoter region in pMU2385, Tp^R^	This study
*rnr-1-lacZ* (B)	*rnr-1* promoter region in pMU2385, Tp^R^	This study
*etpB-lacZ* (A)	*etpB* promoter region in pMU2385, Tp^R^	This study
*etpB-lacZ* (B)	*etpB* promoter region in pMU2385, Tp^R^	This study
ETEC_3214*-lacZ* (A)	ETEC_3214 promoter region in pMU2385, Tp^R^	This study
ETEC_3214*-lacZ* (B)	ETEC_3214 promoter region in pMU2385, Tp^R^	This study


**Table 2:** Primers used in this study.

	Sequence 5′–3′
cfaDkoF	ATCAGAAAGGCCATATGTTGCATTCAGATTGAACGGAGATATACTAAGGCTGTGTAAGCTGGAGCTGCTTC
cfaDkoR	GAATTTTCAAGTAGTAATAACGCAGCCTTGCTCATTCTTAATTGCATTAAAATCGGTCCATATGAATATCCTCCTTAG
pKD4Fs	TGACGAGTTCTTCTGAGCGGGAC
pKD4Rs	TCTAGCTATCGCCATGTAAGCC
cfaDseqF	CAGACTTGTATTTCTTGAAAGAGGAG
cfaDseqR	AGCTGGTCCTGATGCATG
CfaDHindIII	AAGCTTGCAGTATTATGATGCTACTATTTAACACTC
CfaDSalI	GTCGACAACAATATTGGCGCTATTACGCGCC
MBPCfaD-F	GGATCCATGGATTTTAAATACACTGAAGAAAAAGAAATGATAAAAA
MBPCfaD-R	AAGCTTCAATTCAGTTTGCATCGCAATAAATCTC
cfaABam	GGATCCAACCTTGTAGTGGCGATGAGC
cfaAHind	AAGCTTCCACCATCCCAATTGGGTATATCAAC
rnrForBmA rnrRevXba	AGGATCCTTCAATCTGAATGCAACATATGGCC TCTAGACTAATCCATGTTTTTTTCTGCCAGCGC
rnrForB rnrRevB	CTTTTTATTATTATTATTATTTTGGGTCTTTGGTGCCTTGCTGTG CAAAAAAAGTTGAAAAAACATATCTTTCTTTTAGCGAAAG
rnrRevXb	ATCTAGACTAAATCCATGTTTTTTTCTGCCAGC
rnrEMSA-F	ATCCATCTTATACCTACAAAAGGCAAGAG
rnrEMSA-R	GTGATGCTTGTTTCCAAAGTTCAAAGGCAG
rnrPx	GTTTTTTTCTGCCAGCGCGGC
EtpBBamA EtpBForB EtpBRevB	AGGATCCGGGTTATCGCTCGCCACGGG TGCTTTATTTCCCATTAATATTGCTGTTATATGACTTTC CAACTCACTTCGGAGGCT
EtpBHind	GAAGCTTGCTCCAGCATCTGGAGACG
EtpBPx	GAACGGTTCTGACATGAATTTCACCACC
etpBEMSA-F	GGGTTATCGCTCGCCACGGG
etpBEMSA-R	GAACGGTTCTGACATGAATTTCACCACCA
3214BamA	AGGATCCGGTGGCGGGGGATTCACAACG
3214ForB 3214RevB	AATGTCTTTCCCCAAATTTTTGACTTAATCTTAAATAAG CTATTAATCTTACATGAATAGAGC
3214Hind	GAAGCTTGCGATATGTTGATATAAATCAAACACTCCG
3214Px	ATCCAAGAACAGCAAGAATACCACTC
3214EMSA-F	GGTGGCGGGGGATTCACAACG
mtrEMSA	CTGTCTTTTGTACTCGTGTACTGGTACA
mtrEMSA-R	GCCGGAATGCAGCATACAGAACC


### DNA Manipulation Techniques

Restriction enzyme digestions were performed using enzymes and buffers from New England BioLabs (NEB) according to the manufacturer’s instructions. DNA sequencing was performed using the BigDye terminator (v3.1) cycle sequencing kit (Applied Biosystems) in accordance with the manufacturer’s instructions. Sequencing reactions were completed in a GeneAmp PCR system 9700 thermal cycler (Applied Biosystems). Analysis of sequencing results was achieved using the Sequencher (Gene Codes) and DNA Strider^1^ programs. PCR amplifications were performed using GoTaq Green Master Mix (Promega), or Phusion Flash High-Fidelity PCR Master Mix (Finnzymes). PCR primers (**Table 2**) were obtained from GeneWorks (Australia) or Bioneer Pacific. To construct the plasmids used in this study, we first cloned the various PCR fragments into pCR2.1-TOPO (Invitrogen/Life Technologies) or pGEM-T Easy (Promega). Following sequence verification, we cloned the various inserts from the pCR2.1-TOPO or pGEM-T Easy derivatives into the appropriate vectors (**Table 1**). To scramble the CfaD boxes in the regulatory regions of the *rnr-1, etpB*, ETEC_3214 genes, we used the Q5 site-directed mutagenesis kit (New England Biolabs).

### Construction of Δ*cfaD* and Δ*cfaABCE* Knockout Mutants of *E. coli* H10407

The λ Red recombinase system (Datsenko and Wanner, 2000) was used to construct a *cfaD* knockout mutation in ETEC strain, H10407. First, the Phusion high-fidelity DNA polymerase, the primer pairs, cfaDkoF/cfaDkoR, and plasmid, pKD4, were used in a PCR reaction to generate a DNA fragment that contains the kanamycin-resistance gene cassette (Kan^R^) flanked by 50-bp DNA sequences corresponding to the upstream and downstream regions of the *cfaD* gene. This linear DNA fragment was then transformed by electroporation into *E. coli* H10407, which carried plasmid pKD46, encoding the λ Red recombinase system. The resultant Δ*cfaD*::kan^R^ mutant was confirmed by PCR using primer pairs pKD4Fs/CfaDseqR and pKD4Rs/CfaDseqF.

The same method was also used to construct Δ*cfaABCE*::kan mutant except that primers cfaAkoF and cfaEkoR were used to generate the Kan^R^ flanked by DNA sequences of upstream and downstream regions of the *cfaABCE* gene cluster. Primer pairs cfaAseqF/pKD4Rs, and pKD4Fs/cfaEseqR were used to confirm the Δ*cfaABCE*::kan mutation in H10407.

### RNAseq Analysis

Overnight cultures of *E. coli* H10407Δ*cfaD*(pACYC184) and H10407Δ*cfaD*(pACYC184-CfaD) were diluted 1 in 100 in LB with 25 μg/ml chloramphenicol, with or without 45 mM bicarbonate, and incubated at 37°C with shaking to an OD_600_ of approximately 0.8. Two volumes of RNAprotect (Qiagen) were added to one volume of culture, and the samples were incubated at room temperature for 10 min. They were then centrifuged at 4000 × *g* for 20 min. RNA was extracted using the FastRNA Pro Blue Kit (QBiogene) according to the manufacturer’s instructions, except that after the addition of chloroform, 350 μl of the upper phase were added to 35 μl sodium acetate in 875 μl cold 100% ethanol, and held at -20°C overnight. The samples were then centrifuged at 4000 × *g* for 15 min at 4°C, after which the supernatants were removed and the pellets were left to air dry for approximately 45 min before being resuspended in 87.5 μl nuclease-free water. Samples were treated with 2.5 μl DNase I and 10 μl RDD buffer from the RNase-Free DNase Set (Qiagen). Following a purification using the RNeasy MinElute cleanup kit (Qiagen), the samples were eluted in 34 μl of RNase-free water. RNA quality and integrity were examined using an Agilent Bioanalyzer before subjecting samples to rRNA depletion by using the Ribo-Zero^TM^ Magnetic Kit (Gram-negative bacteria) (Epicenter) according to the manufacturer’s instructions. The samples were then re-purified using the RNeasy MinElute cleanup kit (Qiagen) with a final elution volume of 12 μl in RNase-free water. RNA sequencing was performed at the Australian Genome Research Facility using an Illumina Hiseq 2000.

Raw data file reads were subjected to trimming of low-quality bases and removal of adapter sequences using Trimmomatic (v0.30) (Lohse et al., 2012). Trimmed reads were aligned to the H10407 genome (NCBI accession NC_017723.1) using Bowtie (Langmead et al., 2009). SAM files produced by Bowtie were converted to BAM files and coverage depth was calculated using SAMtools (Li et al., 2009) resulting in >98% coverage across the genome with an average of 172× coverage (ranging between 110× and 211× depending on the sample). Aligned reads were then counted per gene in the ETEC H10407 genome using the HTSeq software suite. Data were analyzed by using the SPARTA program (Johnson et al., 2016). Differentially expressed genes were identified as those with an average normalized count >100, differential gene expression of >4.5-fold, and a *P*-value of <0.05. **Supplementary Table S1** contains all the differential gene expression data generated in this study.

### β-Galactosidase Assay

To examine the promoter functions and to evaluate the effect of the small molecule inhibitors on CfaD-mediated activation, cells were grown to mid-log phase (OD_600_∼0.6), with or without shaking, respectively, after which β-galactosidase activity was assayed as described by Miller (1974), with specific activity expressed in units as described therein. Data are the means ± SD of at least three independent assays.

### Expression and Purification of MBP::CfaD

A DNA fragment, which contained the coding region of *cfaD* and was flanked by HindIII and BamHI sites, was amplified by PCR using primer pairs MBPcfaD-F and MBPcfaD-R and genomic DNA from *E. coli* H10407. The amplified DNA fragment was cloned into TOPO-TA and sequenced. The *cfaD* fragment was then excised and cloned into the HindIII and BamHI sites of pMAL-c2x (NEB) to create a fusion with the C-terminal end of the *malE* gene. The MBP::CfaD fusion protein was overexpressed and purified by using the methods described by the manufacturer. The concentration of purified MBP::CfaD protein was determined by using the Bradford method (Bradford, 1976).

### Electrophoretic Mobility Shift Assay (EMSA)

The four ^32^P-labeled PCR fragments used in the EMSA were generated as follows. The primers rnrEMSA-R, etpBEMSA-R, 3214Px and mtrEMSA-R (**Table 2**) were labeled with ^32^P at their 5′ end by using [γ-^32^P]-ATP and T4 polynucleotide kinase. The DNA fragments containing the promoter regions of *rnr*, *etpB*, ETEC_3214 and *mtr* were each generated by PCR using primer pairs ^32^P-rnrEMSA-R/rnrEMSA-F, ^32^P-etpBEMSA-R/etpBEMSA-F, ^32^P-3214Px/3214EMSA-F or ^32^P-mtrEMSA-R/mtrEMSA-F, respectively, with TOPO-TA carrying the *rnr*, *etpB*, ETEC_3214 or *mtr* regulatory regions as template. Each end-labeled fragment was incubated with varying amounts of purified MBP::CfaD protein at 37°C for 30 min in the binding buffer (10 mM Tris⋅HCl [pH 7.4], 45 mM NaHCO_3_, 50 mM KCl, 1 mM DTT, 100 μg/ml BSA, and 5 ng/μl poly(dI-dC)). Glycerol was added to a final concentration of 6.5%. Samples were analyzed by electrophoresis on 5% native polyacrylamide gels (37.5:1). Electrophoresis was carried out at 4°C for approximately 12 h at 10 V/cm.

### Primer Extension Assay

Primer extension was performed as follows. Total cellular RNA was purified from *E. coli* MC4100 derivatives containing pACYC184-CfaD and *rnr-1-lacZ-A, etpB-lacZ-A*, ETEC_3214*-lacZ-A* or pMU2385. Cells were grown to OD_600_∼0.8, and RNA was isolated by using the FastRNA Pro kit (MP Biomedicals) and RNA Miniprep Kit (Qiagen). ^32^P-labeled primers; ^32^P-rnrPx, ^32^P-EtpBPx and ^32^P-3214Px, were used to probe the start sites of transcription of the *rnr*, *etpB* and ETEC_3214 promoters, respectively. Each labeled primer was co-precipitated with 5 μg of total RNA. Hybridization was carried out at 45°C for 15 min in 10 μl of Tris-EDTA (TE) buffer containing 150 mM KCl. Primer extension reactions were started by the addition of 24 μl of extension solution (20 mM Tris-HCl [pH 8.4], 10 mM MgCl_2_, 10 mM dithiothreitol [DTT], 2 mM deoxynucleoside triphosphates [dNTPs], and 1 U/μl avian myeloblastosis virus [AMV] reverse transcriptase) and were carried out at 42°C for 60 min. Samples were precipitated and then analyzed on a sequencing gel. GA ladders were generated by using the one-step method (Song et al., 1997).

### Screening Assay for Small Molecule Inhibitors of CfaD

An overnight culture of test strain MC4100(*cfaA-lacZ*, pACYC184-CfaD) was diluted 1 in 100 in LB containing 45 mM NaHCO_3_ and then was dispensed in 100 μl volumes into 96-well microtiter trays. Compounds (5 μl, 2 mM) from the Chembridge Microformats library (ChemBridge Corp.) were added to the wells in columns 2–11 of these test plates. The wells in columns 1 (used to determine the mean luminescence signal from untreated cells) and 12 (background) received 5 μl of 100% DMSO alone. Control plates were filled with the same volume of compounds or DMSO alone and the control strain, *E. coli* JP8042(*mtr-lacZ*, pACYC177-TyrR), diluted (1:100) in LB broth containing 1 mM tyrosine. The *mtr-lacZ* fusion is a house-keeping gene promoter reporter that is activated by TyrR (Yang et al., 1993). The inclusion of this control strain in the assay allowed us to filter out false-positives, such as compounds that inhibited bacterial growth or the β-galactosidase enzyme.

All samples were incubated at 37°C for 18 h, after which 8 μl of lysozyme (Sigma) solution (6 mg/ml) was added to the wells, followed by incubation at room temperature for another 20 min. The β-galactosidase released from the bacterial cells was converted to a luminescence signal by adding 25 μl of Beta-Glo (Promega) solution into the wells of columns 1–11. The level of luminescence from each well was measured 1.43, 2.86, and 4.3 min later using the FLUOStar Omega plate reader (BMG Labtech).

### Computational Modeling and Docking

HHpred, a homology detection program which creates Hidden Markov Models (Soding et al., 2005), detected the ToxT crystal structure (PDB code 3GBG) (Lowden et al., 2010) as the closest homolog to the amino acid sequence of CfaD. A PIR alignment was generated from this crystal structure and submitted to MODELLER v 9.10 (Sali et al., 1995). The resulting CfaD homology model was then minimized sequentially (hydrogens, then side chains, and then main chains) under the Merck Molecular Force Field for 10,000 iterations and assessed using Procheck (Laskowski et al., 1993), which found that 99% of the residues resided in the allowed regions of the Ramachandran plot. Chi1–Chi2 plots, main-chain parameters, side-chain parameters, G-factors, bond angles, and bond lengths were also all within the allowed parameters.

A SiteID search was conducted within Sybylx2.1 (Certara L.P.) to look for potential compound-binding pockets in the CfaD model. Docking protomols were created for each potential compound-binding pocket in Surflex, Sybyl 2.1 (Certara L.P.). For all the protomols the threshold was reduced to 0.32 and the bloat increased to 2 Å. The CfaD inhibitors, CH-1 and CH-2 were then docked into each protomol using the Surflex-Dock Geom mode in Sybylx2.1 (Certara L.P.). Flexibility of rings was allowed, but all other parameters were kept at default values. The top 30 scored solutions were retained and analyzed visually.

We have assembled a library of approximately 10 million commercially available compounds. Unity (Sybylx2.1, Certara L.P.) was used to conduct 2D analog searches of the hit compounds using a Tanimoto similarity of greater than 65%. Compounds were then purchased and assayed. All figures were constructed using the PyMOL Molecular Graphics System, Version 1.8 Schrödinger, LLC.

### Analysis of CFA/I Fimbriae Production by H10407 in the Presence or Absence of CfaD Inhibitors

To extract bacterial surface proteins, *E. coli* H10407 and its derivatives were grown in 10 ml CFA medium (Evans et al., 1979) in the absence or presence of CfaD inhibitors (25 μM or 50 μM), CH-1 and CH-2, at 37°C for 6 h with shaking at 100 rpm. Cells were harvested by centrifugation at 3,000 × *g* for 10 min, resuspended in 250 μl of phosphate-buffered saline (pH 7.4), vortexed at high speed for 1 min and subsequently incubated at 60°C for 20 min with intermittent vortexing. The samples were then pelleted by centrifugation at 3,000 × *g* for 10 min, and the supernatant was transferred to a fresh tube, where it was mixed with NuPAGE lithium dodecyl sulfate sample reducing buffer (Thermo Scientific) and heated at 70°C for 10 min. The samples (25 μl) were then separated by SDS-PAGE using 12% Bis-Tris NuPAGE gels (Invitrogen), and the separated proteins were stained with Coomassie brilliant blue G250. The bands of interest were excised, trypsin-digested, and analyzed by tandem mass spectrometry at the Proteomics Laboratory, Walter and Eliza Hall Institute of Medical Research, Melbourne, VIC, Australia.

## Results

### Transcriptomic Analysis of CfaD-Mediated Gene Regulation

To measure differential gene expression in response to CfaD and screen the *E. coli* H10407 genome for previously unidentified genes that are regulated by CfaD, RNAseq transcriptional profiling was performed on two H10407 *cfaD* knockout mutants that carried either the control plasmid, pACYC184, or the CfaD-complementing plasmid, pACYC184-CfaD. Both CfaD homologs, RegA and ToxT, respond to bicarbonate ions in transcriptional activation of their target promoters (Abuaita and Withey, 2009; Yang et al., 2009). Accordingly, we tested the effect of bicarbonate ions on CfaD-mediated activation by growing the H10307 derivatives in LB in the absence or presence of 45 mM sodium bicarbonate.

**FIGURE 1:**
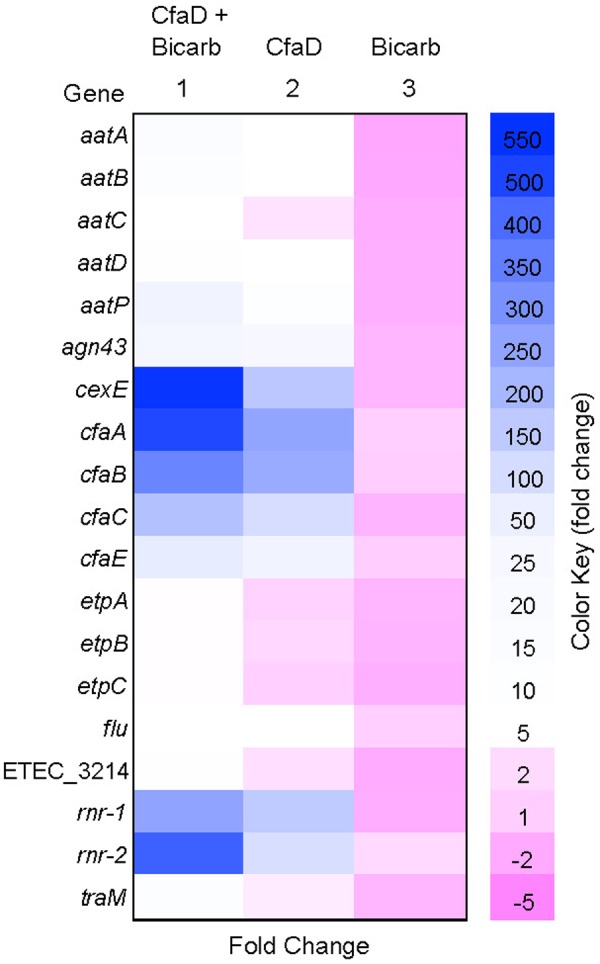
Heat map showing the effects of CfaD and sodium bicarbonate on the expression of enterotoxigenic *Escherichia coli* (ETEC) genes. RNASeq data were used to determine fold changes in gene expression by comparing the number of transcripts from the following *E. coli* derivatives: H10407 CfaD^+^ grown with bicarbonate (column 1); H10407 CfaD^+^ without bicarbonate (column 2), and H10407 CfaD^-^ with bicarbonate (column 3), to those from H10407 CfaD^-^ without bicarbonate. Negative and positive values represent the degrees of down- and up-regulation, respectively.

The results showed that the transcription of 19 genes in 10 operons was markedly activated (between 5- and 550-fold) by CfaD in the presence of sodium bicarbonate (**Figure 1**). Three known targets of CfaD, the *cfaABCE* and *cexE-aatPABCD* clusters, encoding CFA/I fimbriae, Aap dispersin-like protein and its transport system, respectively, were highly upregulated by CfaD. We also identified an additional 10 transcriptional units that were positively regulated by CfaD. These included two copies of the *rnr* gene (*rnr-1* and *rnr-2*) encoding an Rns/CfaD negative regulator that is present in many Gram-negative bacterial pathogens (Santiago et al., 2014); two copies of a gene encoding Antigen 43, annotated *agn43* and *flu* (Owen et al., 1987; Crossman et al., 2010); the gene cluster *etpBAC* encoding an extracellular bridging adhesin (Roy et al., 2009); and the *traM* gene whose product is required for conjugation by IncF group plasmids (de la Cruz et al., 2010). An open reading frame, ETEC_3214, encoding a protein of unknown function was also strongly upregulated (**Figure 1**).

In the CfaD^-^ background, sodium bicarbonate had no effect on the expression of the CfaD-activated genes. In the CfaD^+^ strain, however, transcription of these genes was significantly enhanced by sodium bicarbonate (up to 4.6-fold; **Figure 1**). These results suggested that *E. coli* H10407 may use CfaD to sense and respond to bicarbonate ions in the small intestine, leading to increased levels of virulence gene expression.

### Further Analysis of Selected CfaD Target Promoters

**FIGURE 2:**
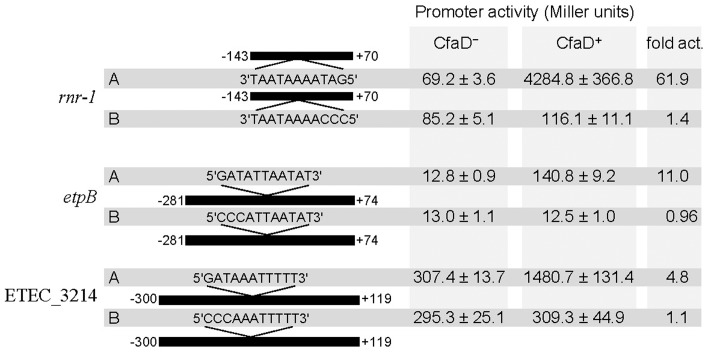
β-galactosidase expression by constructs A and B of the *rnr-1*, *etpB*, and ETEC_3214 promoter-*lacZ* fusions in CfaD^-^ and CfaD^+^ backgrounds, MC4100(pACYC184) and MC4100(pACYC184-CfaD), respectively. Numbering of the various promoter fragments is relative to their start site of translation. The sequences of CfaD boxes in constructs A of *rnr-1*, *etpB*, and ETEC_3214 and the base changes introduced into the CfaD boxes in constructs B are shown. β-galactosidase activity is expressed as Miller units, and the values are means plus standard deviations from three independent assays. The fold activation (fold act.) is the ratio of β-galactosidase activity of the CfaD^+^ strain to that of the CfaD^-^ strain.

Analysis of the sequences of the newly identified CfaD target genes listed in **Figure 1** using the Rns-binding site consensus sequence (Munson and Scott, 2000; Tan et al., 2011) revealed the likely CfaD-binding sites within the vicinity of the promoter regions of the *rnr-1* (and its copy, *rnr-2*), *etpBAC* and ETEC_3214 transcriptional units. To determine if these putative binding sites are required for CfaD-mediated activation, we made two promoter-*lacZ* fusions for each of three transcriptional units: *rnr-1*, etpBAC and ETEC_3214 (**Figure 2**). In each pair, the *lacZ* fusion carrying the wild-type promoter fragment (construct A in **Figure 2**) contained a putative CfaD-binding site and the *lacZ* fusion carrying the mutant promoter fragment (construct B) had this sequence scrambled (**Figure 2**).

β-galactosidase assays showed that CfaD positively controls the expression of the three construct A promoters (**Figure 2**). In these cases, the *rnr-1, etpB* and ETEC_3214 promoters were upregulated 62-, 11-, and 5-fold, respectively, by CfaD. In contrast, the three construct-B promoters exhibited various basal levels of transcription that were not enhanced by CfaD (**Figure 2**). These results are consistent with the hypothesis that the putative CfaD-binding sites identified in the promoter regions of these three operons are required for activation by CfaD. The identification of the three new CfaD-binding sites allowed us to update the consensus sequence (**Figure 4C**).

### Direct Interaction of CfaD with the Regulatory Regions of *rnr-1, etpB*, and ETEC_3214

**FIGURE 3:**
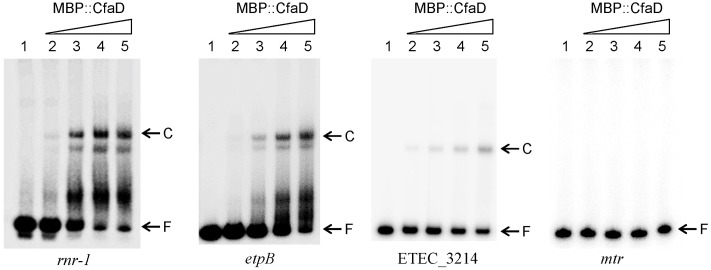
Electrophoretic mobility shift assay demonstrating the binding of MBP::CfaD to the *rnr-1*, *etpB*, and ETEC_3214 promoters. Each ^32^P-labeled PCR fragment was incubated for 30 min at 30°C with increasing amounts of MBP::CfaD (0, 37.5, 75, 150, or 300 nM MBP::CfaD in lanes 1–5, respectively), after which the samples were analyzed on native polyacrylamide gels. Bands of free DNA (F) and the major DNA-protein complexes (C) are indicated at the right of the gels. The *mtr* promoter fragment was used as a negative control in this assay.

To test if CfaD binds directly to the regulatory regions of *rnr-1, etpBAC*, and ETEC_3214, we performed electrophoretic mobility shift assays (EMSA). Previous studies have shown that CfaD is insoluble in water (Munson and Scott, 1999). To overcome this problem, we used a purified fusion protein (MBP::CfaD) for these assays. The three DNA fragments spanning the regulatory promoter-operator regions of *rnr-1, etpB*, and ETEC_3214, as well as a control fragment carrying the *mtr* regulatory region, were each end-labeled with ^32^P and incubated with various concentrations of MBP::CfaD. The samples were then analyzed on native polyacrylamide gels. As shown in **Figure 3**, a concentration-dependent formation of CfaD-DNA complexes was detected with *rnr-1, etpB*, and ETEC_3214 promoter fragments, but not with the control DNA. These results showed that CfaD activates these three gene targets through a direct interaction with their respective regulatory regions.

### Identification of Transcriptional Start Sites of *rnr-1*, *etpB*, and ETEC-3214

**FIGURE 4:**
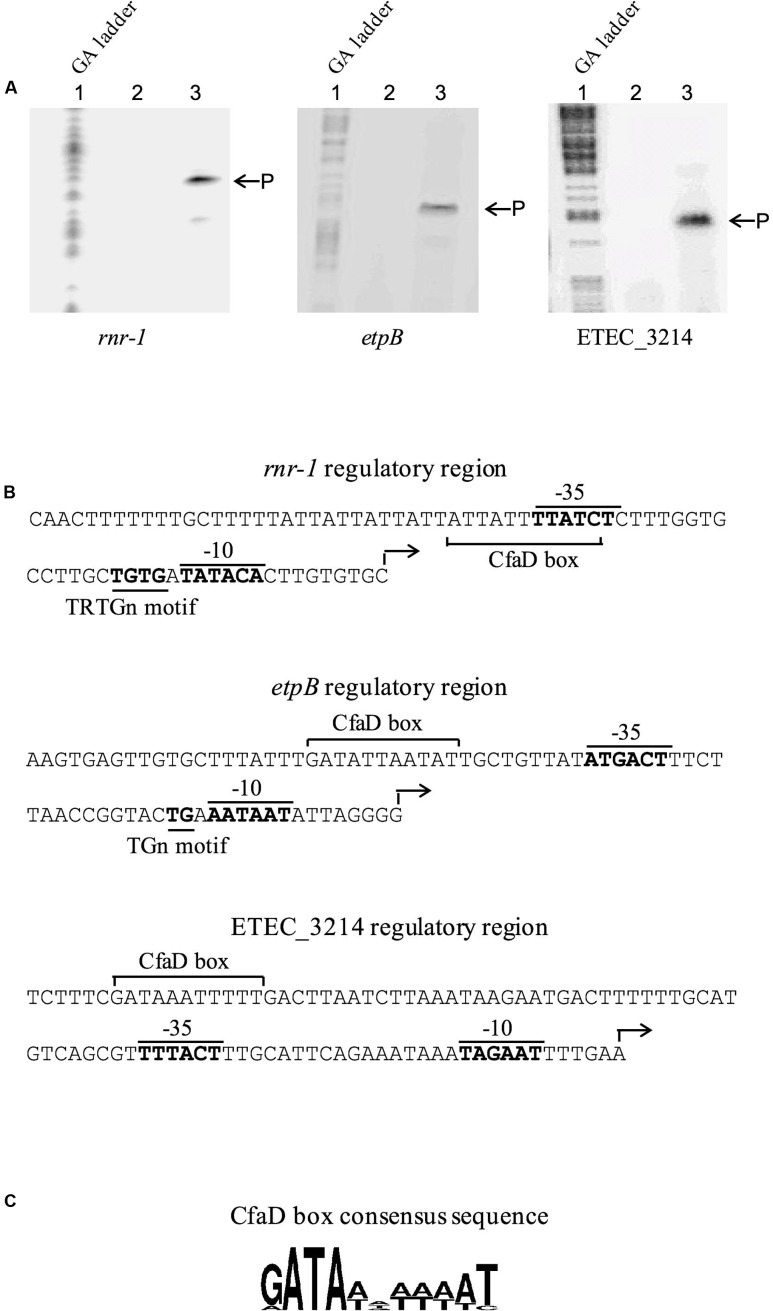
Mapping the start site of transcription of the *rnr-1*, *etpB*, and ETEC_3214 promoters by primer extension and the nucleotide sequence of the corresponding regulatory regions. **(A)** RNA of each test sample was isolated from *E. coli* MC4100(pACYC184-CfaD) with the A construct of *rnr-1-*, *etpB-*, or ETEC_3214-*lacZ* fusion (**Figure 2**). The control RNA sample was obtained from *E. coli* MC4100(pACYC184-CfaD, pMU2385). Lane 1, GA ladder. Lane 2, control sample. Lane 3, test sample. P shows the positions of the extension products. **(B)** The nucleotide sequences of the *rnr-1*, *etpB*, and ETEC_3214 regulatory regions. The start sites of transcription are indicated by an angled arrow and the putative -10 and -35 regions, the TRTGn and TGn motifs and the putative CfaD-binding sites (CfaD box) are labeled. **(C)** The consensus sequence of the CfaD-binding sites. The consensus is derived by using the sequences of the CfaD-binding sites identified from previous and current studies (Munson and Scott, 1999; Pilonieta et al., 2007) and the WebLogo sequence generator application (Crooks et al., 2004).

The transcriptional start sites associated with the *rnr-1, etpB*, and ETEC_3214 promoters, were determined by using primer extension. Briefly, three derivatives of *E. coli* MC4100 carrying pACYC184-CfaD and construct A of *rnr-1-lacZ, etpB-lacZ* or ETEC_3214*-lacZ* (**Figure 2**) were grown to mid-log phase, after which total RNA was extracted. ^32^P-labeled primers were then used to probe the transcriptional start sites of the three transcriptional units. The results showed that in each case one major extension signal was observed (**Figure 4A**), indicating that these three operons are each driven by a single promoter. The transcriptional start sites corresponded to positions 31-, 108-, and 61-bp upstream of the start codons of the *rnr-1, etpB* and ETEC-3214 coding sequences, respectively. Based on the position of the start sites of transcription, putative -35 and -10 regions of the three promoters were identified (**Figure 4B**).

### Screening for Chemical Inhibitors of CfaD

To investigate the feasibility of CfaD as a drug target, we screened the Chembridge Microformat library (ChemBridge Corp.) for small molecule compounds that inhibited the ability of CfaD to activate expression of the *cfaA* promoter. After screening approximately 20,000 compounds, we identified 63 compounds that showed various degrees of inhibition on CfaD-mediated activation of *cfaA* expression. However, following further tests, only one compound, which we named CH-1, reproducibly exhibited a complete inhibition of CfaD function at the concentration of 100 μM.

**FIGURE 5:**
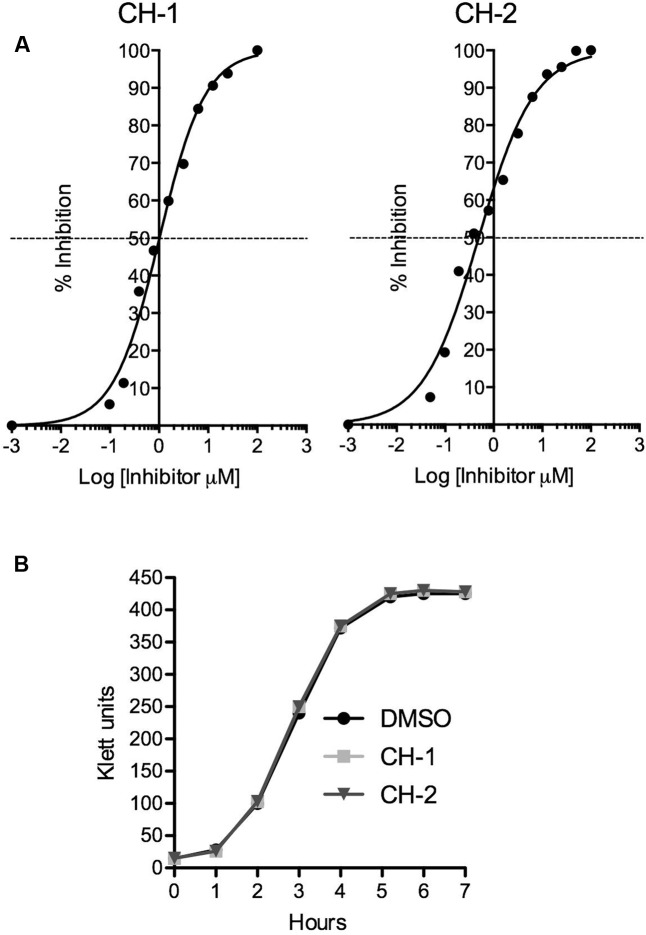
Potency of small molecule inhibitors of CfaD. **(A)** The IC_50_ values of CH-1 and CH-2 were determined using dose-response curves. Data were obtained by measuring the β-galactosidase activities of the *E. coli* strain MC4100(*cfaA-lacZ*, pACYC184-CfaD) grown in the presence of varying concentrations of CH-1 and CH-2, and then used to calculate the IC_50_ (Sigmoidal dose-response equation, GraphPad Prism 5). **(B)** *E. coli* H10407 was grown at 37°C in LB broth with shaking (250 rpm) in the absence or presence of CH-1 or CH-2 (100 μM). Bacterial growth was followed using a Klett colorimeter over a period of 6 h.

Using CH-1, we then computationally screened our in-house virtual library of ∼10 million chemicals for 2-D analogs of CH-1. Thirty-two analogs were identified and tested for their ability to inhibit CfaD activity at the *cfaA* promoter. We identified several compounds with varying activity, but only one of these analogs, which we named CH-2, exhibited greater potency than CH-1. As shown in **Figure 5A**, the IC_50_ concentrations for CH-1 and CH-2 are 1.02 and 0.49 μM, respectively. To verify that these two compounds did not inhibit bacterial growth, *E. coli* H10407 was grown in the absence or presence of CH-1 and CH-2 (100 μM), and no differences in growth or viability were observed (**Figure 5B**).

**FIGURE 6:**
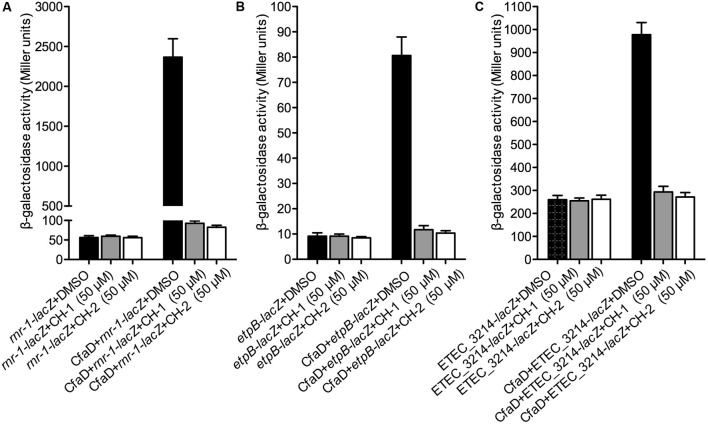
Inhibition of CH-1 and CH-2 on transcription of the *rnr-1*, *etpB*, and ETEC_3214 promoters. The *E. coli* MC4100 strains used in this analysis all contain a pair of plasmids comprising a promoter-*lacZ* transcriptional fusion with either pACYC184 or pACYC184-CfaD. β-galactosidase assays were performed to evaluate the effect of CH-1 and CH-2 on CfaD-mediated transcriptional activation of the *rnr-1* **(A)**, *etpB* **(B)**, and ETEC_3214 **(C)** promoters.

We next examined the effect of CH-1 and CH-2 on CfaD-mediated activation of the *rnr-1*, *etpB* and ETEC-3214 promoters. *E. coli* MC4100 strains carrying construct A of *rnr-1-lacZ, etpB-lacZ* or ETEC_3214*-lacZ* (**Figure 2**) and either pACYC184 (control) or pACYC184-CfaD (test) were grown in the absence or presence of CH-1 and CH-2 (50 μM), following which β-galactosidase assays were performed. Data in **Figure 6** showed that, while neither of the inhibitors had any effect on the basal level transcription of the three promoters in the CfaD^-^ background (control), both CH-1 and CH-2 strongly inhibited CfaD-mediated activation of the three promoters in the CfaD^+^ background (test).

### Characterization of the Interaction of CH-1 and CH-2 with CfaD

**FIGURE 7:**
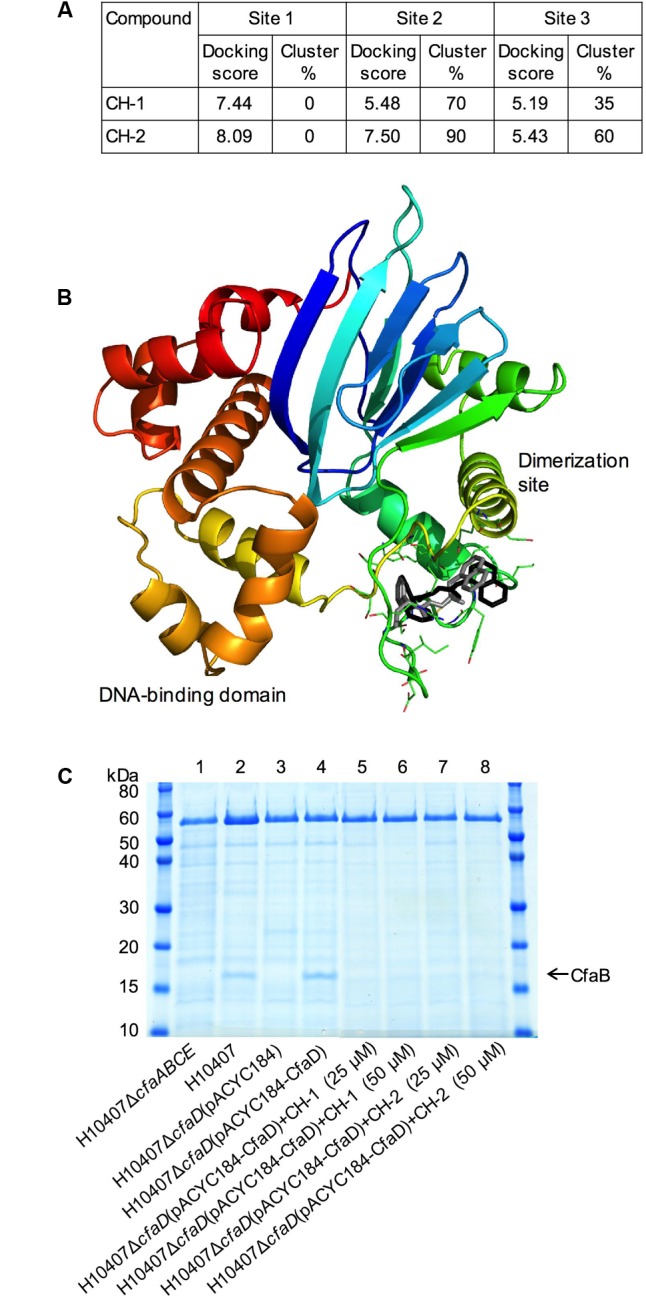
Characterization of the interaction of CH-1 and CH-2 with CfaD and confirmation of the inhibitory activity of CH-1 and CH-2 on the production of the CFA/I fimbriae in *E. coli* H10407. **(A)** Table showing the docking scores and clustering values for the top docked solutions of CH-1and CH-2. **(B)** A docking model showing the proposed interaction of CH-1 (black) and CH-2 (gray) with the CfaD protein (ribbon). The homology model of CfaD was generated from the crystal structure of ToxT of *Vibrio cholerae* and is colored rainbow from N-terminus (blue) to C-terminus (red). The proposed dimerization site (green helix) and DNA-binding domain (red and orange helices) are labeled. **(C)** SDS-PAGE analysis of heat-extracted surface proteins from *E. coli* H10407 and its derivatives demonstrates that the CfaD-dependent production of the major subunit of the CFA/I fimbriae (CfaB) can be inhibited by the presence of CH-1 and CH-2 (25 μM or 50 μM) in growth medium.

To explore the interactions of CH-1 and CH-2 with CfaD, we performed an *in silico* docking experiment. A putative 3-D model of CfaD was generated based on the crystal structure of the ToxT protein of *V. cholerae* (Lowden et al., 2010). We used this model to identify three potential ligand-binding pockets. These sites were all over 10 Å^3^ and accessible to the surface of the protein. CH-1 and CH-2 were docked into all three pockets. Analysis of the generated docking scores (related to the binding energy of interaction) and degrees of clustering in each pocket (a measure of the likelihood of a specific orientation) (**Figure 7A**) favored one of the pockets (pocket 2) over the rest, and correlated with the greater potency of CH-2 (**Figure 5A**). This pocket is located between the putative dimerization site and the DNA-binding domain, and contains residues Lys85, Ile90, Ile91, Tyr92, Gly93, Met94, Ser95, Ile97, Asp98, Thr99, Arg103, Glu145, Glu148, Ile154, Ile156, Ser157, and Ser158 (**Figure 7B**). Both CH-1 and CH-2 were found to cluster predominantly in this pocket, with a similar orientation and convincing docking scores (**Figure 7A**).

### Confirmation of the Inhibitory Activity of CH-1 and CH-2 on Production of the CFA/I Fimbriae in *E. coli* H10407

To examine effect of the inhibitors, CH-1 and CH-2, on CfaD-mediated expression of the CFA/I fimbriae in *E. coli* H10407, we carried out a proteomic analysis using the following *E. coli* strains: wild-type H10407, H10407Δ*cfaD*(pACYC184-CfaD) and two negative controls, H10407Δ*cfaABCE* and H10407Δ*cfaD*(pACYC184). Analysis of heat-extracted surface proteins using SDS-PAGE showed that a protein of approximately 17-kDa was produced in the H10407 and H10407Δ*cfaD*(pACYC184-CfaD) strains, but not in the H10407Δ*cfaABCE* and the H10407Δ*cfaD*(pACYC184) strains (**Figure 7C**). Mass spectroscopic analysis revealed that the 17-kDa protein was CfaB, the major subunit of CFA/I fimbriae. These results confirmed the dependency of CFA/I expression on CfaD in H10407. Importantly, the data in lanes 5–8 (**Figure 7C**) demonstrated that the CfaD-dependent production of CfaB in H10407 was strongly inhibited in the presence of CH-1 or CH-2.

## Discussion

Transcriptomic analyses performed in this study revealed the entire set of operons whose expression is activated by CfaD in ETEC strain, H10407. Newly identified CfaD targets included genes encoding bacterial surface proteins (EtpA, the extracellular bridging adhesin, and Antigen 43); virulence transcriptional regulators (Rnr-1 and Rnr-2); a protein responsible for virulence plasmid transfer (TraM); and ETEC_3214, a protein of unknown function. We also demonstrated that bicarbonate ions enhance CfaD-mediated activation of virulence gene expression.

Among the most highly upregulated genes by CfaD were *rnr-1* and *rnr-2*, which are genetically linked with *cfaD* and a pseudogene, *cfaD-2* (which carries a frame-shift mutation in the coding region), respectively. *rnr-1* and *rnr-2* encode small proteins of ∼7.5 kDa that repress the transcription of *cfaD* and one of its target gene, *cexE* (Santiago et al., 2014). Homologs of *rnr-1* also exist in enteroaggregative *E. coli* and *C. rodentium*, in which they repress the expression of AggR and RegA, respectively (Santiago et al., 2014). These proteins are thought to fine-tune the intracellular levels of their cognate master-regulators, CfaD, AggR, and RegA, to allow precise expression of virulence genes in particular environments. A recent study by Santiago et al. (2016) demonstrated that these proteins are anti-activators, which bind to the central linker domain of their cognate master-regulators, disrupting their dimerization function and preventing them from binding to DNA. Our finding that CfaD strongly activates the expression of these repressors suggests that Rnr-1 and Rnr-2 are responsible for preventing runaway production of CfaD in ETEC, especially because CfaD activates its own transcription (Munson and Scott, 2000). In addition, given that Rnr-1 and Rnr-2 suppress virulence gene expression by downregulating *cfaD*, these two negative regulators are required during the late stages of infection to modify the transcription profile of ETEC as it exits from the host intestine to the external environment.

Another new CfaD target we identified was the *etpBAC* operon located on the pCS1 virulence plasmid of H10407 (Fleckenstein et al., 2006; Roy et al., 2009). Roy et al. (2009) have shown that EtpC is involved in glycosylation of EtpA, and that glycosylated EtpA is exported from bacterial cells by EtpB. EtpA interacts directly with highly conserved regions of flagellin (the major subunit of flagella), to form a bridge between flagella and host cell surface receptors (Roy et al., 2009). This flagellin-EtpA surface complex is critical for intestinal colonization by some ETEC strains. Our finding that the expression of the *etpBAC* operon is activated by CfaD further highlights the importance of CfaD in inducing the production of a variety of surface adhesins that allow ETEC to colonize its hosts.

Enterotoxigenic *Escherichia coli*_3214, which we also found to be directly activated by CfaD, encodes a protein of 245 amino acids. Interestingly, ETEC_3214 is located on a genomic island, which encodes the type II secretion system and the SslE protein that are required for secretion of the heat-labile enterotoxin and the formation of biofilms by ETEC, respectively (Tauschek et al., 2002; Baldi et al., 2012). A BLAST search revealed that ETEC_3214 is also present in the uropathogenic *E. coli* strain, CFT073, and the avian pathogenic *E. coli* strain, APEC O2. In addition, a gene encoding a protein that shares 64% amino acid identity with the predicted product of ETEC_3214 was found in the plant pathogen *Erwinia piriflorinigrans* (CFBP5888). A stretch of 23 amino acids at the N-terminus of these proteins is predicted to form a transmembrane domain^2^, but, the function of this protein and its relevance to ETEC virulence are not known.

Transcriptional analyses of the regulatory regions of *rnr-1*, *etpBAC* and ETEC_3214 identified the σ^70^ promoters responsible for the expression of these operons (**Figure 4A**). Based on the consensus sequence of the CfaD-binding sites (Pilonieta et al., 2007), we identified probable CfaD-binding sites (the CfaD boxes) upstream of the promoters of *rnr-1*, *etpBAC* and ETEC_3214 (**Figure 4B**). Mutation of these sequences abolished CfaD-mediated activation of these three operons without affecting their basal levels of transcription (**Figure 2**). Furthermore, we showed using EMSA that CfaD interacts directly with regulatory regions of all three operons (**Figure 3**). The EMSA also showed that CfaD formed multiple complexes with the *rnr-1* and *etpB* promoters but only a single complex with the ETEC_3214 promoter. This indicates the presence of more than one CfaD-binding site in the regulatory regions of *rnr-1* and *etpB*. Nevertheless, our mutational analysis clearly demonstrated that the CfaD boxes located immediately upstream of their respective -35 sequences are responsible for activation by CfaD (**Figures 2**, **4B**). The promoter regions of these operons are AT-rich and predicted to be highly curved by the “bend.it” program^3^, supporting our suggestion that these promoters are bound and silenced by H-NS (Dorman, 2004). Given that the CfaD-binding sites are also located in the curved regions, we believe that the binding of CfaD counteracts the action of H-NS, leading to enhanced transcription.

Because of the central role played by CfaD in the global regulation of virulence gene expression in ETEC, we predicted that chemical inhibition of CfaD would disrupt the ability of the pathogen to cause disease. To explore this possibility we developed an *in vitro* assay to screen for small molecule inhibitors of CfaD. By screening a commercial chemical library and structural analoging, we identified two compounds, CH-1 and CH-2, which inhibited the CfaD-mediated activation of the *cfaA, rnr-1, etpB*, and ETEC_3214 promoters in *E. coli* K-12 and the production of the CfaB protein in *E. coli* H10407 (**Figure 6C**). These inhibitors are specific for CfaD as they did not inhibit the TyrR/*mtr* regulatory system of *E. coli* (Yang et al., 2013). Moreover, data from the computational docking suggested a potential inhibitor binding pocket between the dimerization and DNA-binding domains of CfaD. Although both inhibitors have a high potency *in vitro*, further chemical optimization is required to improve the water-solubility of these compounds before we can assess their efficacy *in vivo* in animals. Nevertheless, our results do prove that, like several other AraC-like virulence regulators, including ToxT, RegA, and VirF (Hung et al., 2005; Koppolu et al., 2013; Yang et al., 2013), CfaD can be exploited as a drug target for the development of novel treatments of ETEC infection.

In summary, in this study we carried out the first transcriptomic analysis of CfaD-mediated regulation in the prototypical ETEC strain, H10407, and identified a number of previously unknown, putative virulence gene targets of CfaD. Molecular characterization of some of these target operons identified the promoters and operators that are responsible for CfaD-mediated activation of gene expression. Furthermore, transcriptional analysis demonstrated that the gut-associated chemical, sodium bicarbonate, acts as a cofactor to enhance expression of the virulence genes controlled by CfaD. The critical importance of CfaD in the control of ETEC virulence makes it a potential target for new types of drugs that could be used to combat ETEC infections (Yang et al., 2013). In this regard, we identified two small-molecule compounds that specifically inhibited the regulatory function of CfaD, demonstrating that this regulator is a suitable target for the development of drugs to combat ETEC infection.

## Author Contributions

CH, JY, MT, and RR-B designed and performed most of the experiments and contributed to the analysis and interpretation of data. DH, KA, QC, and JH carried out some experiments. MT, JH, and MP dealt with the preparation of figures, drafting the work and revising it critically. The manuscript was written by CH, JY, MT, and RR-B and reviewed by all authors before submission.

## Conflict of Interest Statement

The authors declare that the research was conducted in the absence of any commercial or financial relationships that could be construed as a potential conflict of interest.

## Footnotes

1 http://cellbiol.com/soft.htm

2 www.predictprotein.org/home

3 http://pongor.itk.ppke.hu/dna/bend_it.html#/bendit_intro

## Supplementary Material

**TABLE S1 |** RNAseq data generated in this study. The genes in H10407 that are differentially expressed in CfaD-minus and CfaD-plus backgrounds in the presence or absence of sodium bicarbonate.
